# “I Am Not Lonely; I Am Just No Longer Needed”: Unnamed Loneliness, Masculinities, Intimacy, and Mental Health Among Older Men in Lisbon, Portugal

**DOI:** 10.3390/ejihpe16090136

**Published:** 2026-09-08

**Authors:** Henrique Pereira

**Affiliations:** 1 Department of Psychology and Education, Faculty of Social and Human Sciences, University of Beira Interior, Estrada do Sineiro, 6200-209 Covilhã, Portugal; hpereira@ubi.pt; 2 RISE-HEALTH, Department of Psychology and Education, Faculty of Social and Human Sciences, University of Beira Interior, Estrada do Sineiro, 6200-209 Covilhã, Portugal

**Keywords:** loneliness, older men, masculinities, intimacy, mental health, qualitative longitudinal research, Portugal

## Abstract

Loneliness among older men may remain unrecognized when unwanted relational disconnection is not explicitly named as loneliness. This qualitative longitudinal, multimethod study examined how 32 community-dwelling men aged 60 years and older in Lisbon, Portugal, experienced, interpreted, and communicated relational disconnection between January and July 2025. Data were generated through narrative life-history interviews, participant-generated photographic, audio, or written diaries, elicitation interviews, and follow-up interviews. Six interrelated themes were developed and interpreted processually as disruptions and absences, forms of disconnection, identity-protective meaning-making, and responses. The analysis distinguished topics deliberately elicited by the design from empirical refinements and unanticipated temporal or material insights. The four processes initially used to sensitize inquiry—nonrecognition, linguistic substitution, identity protection, and relational concealment—were therefore not treated as discoveries; the data clarified their boundaries, interactions, and reversibility. Four longitudinal patterns were used as non-exclusive interpretive summaries rather than participant classifications. The most distinctive findings were that connection depended not only on contact but on relational consequentiality—being expected, needed, consulted, and able to contribute—and that greater willingness to name loneliness could reflect interpretive change rather than worsening symptoms. Unnamed loneliness was inferred only where participants described unwanted relational disconnection; chosen and satisfying solitude was excluded. Responses should foster emotionally safe, reciprocal, and meaningful relationships while protecting autonomy.

## 1. Introduction

### 1.1. Loneliness as a Multidimensional and Relational Experience

Social connection is a fundamental determinant of healthy ageing and psychological well-being. The World Health Organization estimates that approximately one in six people experience loneliness globally and identifies older adults as a population requiring particular attention (World Health Organization [WHO], 2025). Loneliness is distinct from social isolation: isolation concerns the objective availability of contact and participation, whereas loneliness reflects a painful discrepancy between desired and experienced relationships. Older adults may live alone without feeling lonely or remain profoundly disconnected while living with others or participating regularly in social activities (Hawkley & Cacioppo, 2010; Mansfield et al., 2021; National Academies of Sciences, Engineering, and Medicine [NASEM], 2020).

Persistent loneliness and weak social relationships are associated with mortality, depression, anxiety, sleep disturbance, cardiovascular morbidity, poorer quality of life, and cognitive decline, although these associations are embedded in reciprocal biopsychosocial processes rather than a single inevitable causal pathway (Harrington et al., 2023; Holt-Lunstad et al., 2010, 2015; Luchetti et al., 2024). Loneliness is also multidimensional. Social loneliness concerns insufficient network belonging, emotional loneliness the absence of a close attachment figure, and existential loneliness a more fundamental experience of separateness, emptiness, or disconnection from life (Mansfield et al., 2021; van Tilburg, 2021; Weiss, 1973). These dimensions may diverge in later life: a man may have acquaintances but nobody with whom he can disclose distress, or receive practical assistance without feeling understood, valued, touched, desired, or emotionally safe.

### 1.2. Masculinities, Life-Course Transitions, and Concealed Distress

Gender shapes how loneliness is interpreted, communicated, and measured. Older men do not consistently report greater loneliness than women, yet their lower endorsement of direct loneliness questions may reflect stigma, relationship structures, or reluctance to adopt a category associated with dependency and social failure (Ratcliffe et al., 2024; van Tilburg, 2021). A critical masculinities perspective avoids treating men as homogeneous and instead examines how expectations of autonomy, emotional control, productivity, competence, provision, and usefulness are reproduced, resisted, or reformulated across age, class, sexuality, disability, occupation, and place (Connell & Messerschmidt, 2005). Self-reliance, practical activity, humor, sport, volunteering, and working alongside others can support resilience and companionship, but may not create emotionally safe spaces for grief, fear, shame, or dependency (Nordin et al., 2024). Help-seeking is more likely when support is trustworthy, purposeful, reciprocal, and compatible with a valued identity (Addis & Mahalik, 2003; Seidler et al., 2016; Vickery, 2021).

Later-life transitions can destabilize both relationships and identity. Widowhood may remove companionship, emotional and sexual intimacy, shared routines, and the person who sustained biographical continuity; retirement may remove status, structure, recognition, and activity-based contact; and illness, reduced mobility, caregiving, or dependence may restrict participation and threaten reciprocity (Bennett & Victor, 2012; Botha & Bower, 2024). Distress may consequently be communicated through irritability, withdrawal, poor sleep, anger, somatic complaints, alcohol use, loss of motivation, or perceived burdensomeness rather than explicit loneliness or sadness (King et al., 2020; Wagner & Reifegerste, 2024). Narratives of being unnecessary, having nothing to contribute, or not wanting to trouble others may therefore carry relational and existential meanings even when the loneliness label is rejected.

### 1.3. Unnamed Loneliness, Intimacy, and Diversity

In this study, unnamed loneliness is used as a sensitizing rather than diagnostic concept. It refers specifically to unwanted relational disconnection that a participant communicates without explicitly naming loneliness. Inclusion required narrative evidence of a discrepancy between desired and experienced connection—such as an unwanted absence of intimacy, reciprocity, recognition, reliable disclosure, or a valued relational role—interpreted across the participant’s biography and repeated accounts. Boredom, low mood, grief, purposelessness, irritability, routine, limited contact, or self-reliance were not sufficient by themselves. Experiences were excluded when solitude was described as chosen and satisfying, when no unwanted relational discrepancy was evident, or when an adjacent experience such as grief or role loss was not linked by the participant’s account to a desired relationship or relational position. The four possible processes—nonrecognition, linguistic substitution, identity protection, and relational concealment—were specified before analysis as sensitizing prompts, not as findings to be confirmed. Analysis examined whether, how, and under what conditions the data supported, qualified, or contradicted them.

Network size and contact frequency also provide an incomplete account because loneliness may concern emotional, physical, romantic, and sexual intimacy. Affection, touch, proximity, erotic recognition, and continued visibility as a sexual person can remain important in later life, while widowhood, divorce, partner illness, changes in sexual functioning, disability, and ageism may generate embodied forms of absence (Luhmann & Hawkley, 2016; Sinković & Towler, 2019). Experiences vary further by socioeconomic position, education, migration, ethnicity, health, disability, parenthood, relationship history, and sexual orientation. Gay and bisexual older men may draw on chosen families and friendship networks while also encountering cumulative loss, family estrangement, minority stress, and ageism within LGBTQIA+ communities (Hsieh & Liu, 2021). An intersectional approach is therefore required rather than a universal model based on heterosexual, partnered, able-bodied, or economically secure men.

### 1.4. Evidence Gap and the Present Study

Qualitative research shows that older men may remain silent about loneliness, attempt to “get on with it,” and rely on domestic or activity-based distractions that provide only partial relief (Ratcliffe et al., 2023; Willis & Vickery, 2022). Portuguese evidence has identified family, friendship, health, mobility, community engagement, initiative, and meaningful participation as resources for connectedness among older men (Pereira et al., 2024). The unresolved substantive question is therefore not simply whether older men conceal loneliness, or whether a multimethod design is novel, but what repeated and participant-generated accounts reveal about the mechanism and temporal interpretation of relational disconnection. Existing studies often recruit men who live alone, already identify as lonely, or use community services; prioritize contact over intimacy, touch, usefulness, and existential disconnection; and rely on cross-sectional verbal accounts. The present design was used to test the limits of anticipated ideas and to identify what changed across modes and time: how everyday sensory and material contexts made absence perceptible, how being socially present differed from being consequential to others, and how adopting the loneliness label could change without evidence of parallel symptom deterioration.

The present qualitative longitudinal, multimethod study addressed these gaps by examining how men aged 60 years and older in Lisbon experienced, interpreted, communicated, minimized, and concealed loneliness, relational disconnection, and associated psychological distress. It explored how language and silence, masculine identities, life-course transitions, intimacy, routines, places, objects, health, and social position shaped these experiences, while developing a theoretically informed account of unnamed loneliness. The principal research question was: How do older men experience and communicate forms of loneliness and relational disconnection that they may not explicitly recognize or describe as loneliness?

## 2. Materials and Methods

### 2.1. Study Design

This qualitative longitudinal, multimethod study combined narrative life-history interviews, participant-generated photographic or audio diaries, elicitation interviews, and follow-up interviews. A contextualist epistemology treated accounts as personally meaningful interpretations situated within biographies, relationships, living conditions, health, social identities, and cultural expectations. Critical masculinities and the provisional construct of unnamed loneliness informed the questions asked; accordingly, masculinity, emotion, retirement, intimacy, usefulness, distress, and help-seeking were recognized as elicited domains rather than subsequently presented as wholly spontaneous discoveries. Unnamed loneliness remained a sensitizing rather than diagnostic concept, and chosen solitude, self-reliance, or practical activity were not presumed to indicate concealed loneliness. The analytic contribution was sought in how participants combined, qualified, contradicted, materially represented, and reinterpreted these domains across time. Reporting followed COREQ, where applicable, to this longitudinal and participant-generated design (Tong et al., 2007).

### 2.2. Setting and Participants

The study was conducted from January to July 2025 in community settings in Lisbon, Portugal. Interviews took place in Portuguese in private rooms at community organizations, participants’ homes, or university facilities. The final sample comprised 32 community-dwelling men aged 60–83 years, all of whom completed the study. Eligibility required self-identification as a man, residence in Lisbon, capacity to provide informed consent and participate in an in-depth interview in Portuguese, and experience of changes or challenges involving relationships, roles, routines, belonging, intimacy, usefulness, or emotional well-being. Self-identification as lonely was not required. Exclusion criteria were cognitive or communication difficulties preventing informed consent or meaningful participation, acute instability requiring immediate intervention, or inability to understand the voluntary nature of participation. Mental and physical health conditions were not exclusion criteria when participation could proceed safely. Participant characteristics are summarized in Table 1.

### 2.3. Sampling and Recruitment

Purposive maximum-variation sampling sought diversity in age, relationship history, living arrangement, sexual orientation, education and socioeconomic background, retirement, health and mobility, caregiving, parenthood, community participation, major life transitions, and acceptance or rejection of the loneliness label. Recruitment through community centers, senior universities, parish councils, cultural associations, healthcare centers, retirement organizations, and LGBTQIA+ organizations was adjusted iteratively when perspectives appeared underrepresented. Information power was assessed across the dimensions proposed by Malterud et al. (2016): the study aim was focused; the sample was specific to later-life relational change without requiring loneliness self-identification; each participant contributed repeated, in-depth, multimodal material; the analysis was case-oriented and theoretically informed; and the dialogue was sufficiently detailed to support within-case and cross-case interpretation. These considerations supported adequacy of 32 completed cases for interpretive depth and variation. Recruitment ended when the dataset supported a coherent analysis across relevant contexts, not when an assumed point of code saturation or a numerical code threshold was reached.

Study information described changes in relationships, roles, routines, well-being, and belonging after age 60 rather than recruiting “lonely older men.” Interested men contacted the researcher directly or authorized contact. Written and verbal information covered the study aims, longitudinal commitment, voluntariness, confidentiality and its limits, participant-generated materials, possible discomfort, data management, and withdrawal before written consent was obtained.

### 2.4. Data Collection

Data collection comprised four interconnected stages. First, each participant completed a 75 to 120 min narrative life-history interview beginning with an open invitation to describe important people, relationships, places, and roles. Flexible prompts deliberately addressed family and friendships, masculinity and emotion, work and retirement, bereavement and separation, health and caregiving, community, emotional and bodily intimacy, solitude, usefulness, psychological distress, coping, help-seeking, and the future. These were theory-informed elicitation domains, so their appearance in the Results was not treated as evidence that the domains themselves emerged inductively. The analysis instead examined participants’ meanings, relationships among domains, contradictions, contextual variation, and change across stages. The interviewer distinguished among living alone, social isolation, chosen solitude, loneliness, lack of intimacy, and diminished usefulness, and did not require participants to use the term loneliness.

Second, participants completed a 10- to 14-day photographic, audio, or written diary addressing meaningful places, objects, interactions, difficult times of day, loss, companionship, silence, usefulness, and hope. Third, approximately two weeks later, a 60 to 90 min elicitation interview explored 5–10 participant-selected entries or objects and their histories, emotional meanings, absences, and possible changes in interpretation. Fourth, a 60- to 90-min follow-up interview six to nine weeks later examined continuity and change in relationships, health, routines, intimacy, independence, purpose, support, and self-understanding. Earlier materials were revisited to explore evolving meanings rather than to confirm a single correct interpretation. Participants were instructed not to record identifiable third parties or sensitive documents; alternatives to digital photography or audio were available. Although data collection comprised four procedural stages, longitudinal analysis was organized into three analytical phases: the initial interview, the combined diary and elicitation phase, and the follow-up interview.

### 2.5. Researcher Positioning, Field Notes, and Participant Safety

All interviews, coding, and interpretation were conducted by the author, whose qualitative and sensitive-interviewing experience and professional identity as a psychologist could shape both disclosure and interpretation. Reflexive work therefore focused on three recurrent risks: privileging psychologically legible accounts over practical or culturally specific meanings; reading masculine restraint as deficit; and interpreting adjacent experiences through the pre-existing lens of loneliness. Before and after interviews, the researcher recorded expectations, emotional responses, moments of agreement or surprise, and possible effects of perceived clinical authority. During analysis, these notes were revisited alongside participant accounts; interpretations were tested against chosen-solitude cases, practical forms of intimacy, stable self-reliance, and explanations centered on grief, health, or material constraint. These procedures did not eliminate the single-researcher perspective, but made its influence visible and contestable within the audit trail. Research was clearly distinguished from clinical care. Participants could decline questions, pause, change topic, or stop, and a predefined risk-management protocol guided assessment and referral for current suicidal ideation or serious distress.

### 2.6. Data Recording, Transcription, Translation, and Management

Interviews were digitally recorded with consent, transcribed verbatim in Portuguese, and checked against the recordings. Pseudonyms and study codes replaced identifiers, and potentially identifying contextual details were generalized. Diary files, consent forms, and linking documents were stored separately on encrypted, password-protected institutional systems. Quotations were translated into English by the author and checked against the Portuguese transcripts to preserve conceptual meaning, tone, and colloquial expression.

### 2.7. Data Analysis and Software

Data were analyzed using Braun and Clarke’s reflexive thematic analysis (Braun & Clarke, 2006, 2019, 2021, 2022). A dedicated computer-assisted qualitative data-analysis package such as NVivo or ATLAS.ti was not used. Microsoft Word and Excel were considered adequate for the size, organization, and analytic requirements of the dataset. They supported annotated transcripts, coding tables, participant-level case summaries, longitudinal matrices, theme-development records, and the audit trail. As with any qualitative software, these applications were used for data organization and retrieval rather than for automated interpretation. This approach was selected to maintain close engagement with the multimodal material and preserve case-level, temporal, and biographical coherence; the software supported organization and retrieval but did not automate interpretation.

**Figure 1:**
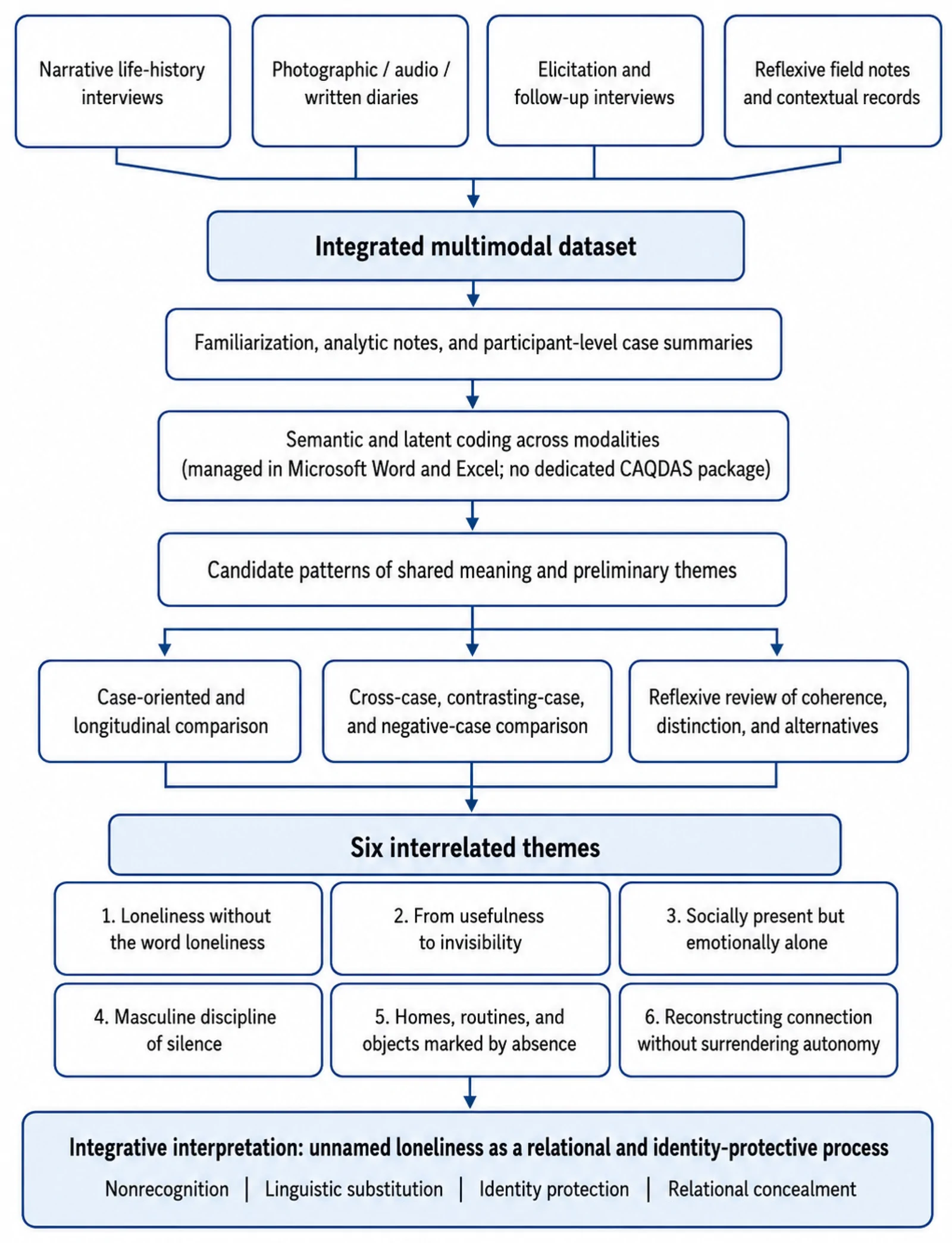
Recursive analytical process and development of the final thematic structure.

Analysis was recursive rather than linear. Familiarization involved repeated reading, selective listening, reviewing photographs and diary entries, and writing analytic notes. Coding addressed the complete dataset at semantic and latent levels and combined inductive engagement with participants’ language and practices with theoretically informed attention to loneliness, masculinities, ageing, intimacy, mental health, and help-seeking. Because codes were repeatedly split, combined, renamed, and reapplied across modalities and time points, a single total of ‘initial codes’ would imply a stability that reflexive thematic analysis did not have. Analytic records instead documented the pathway from annotated extracts to working code clusters, participant-level summaries, candidate organizing concepts, and successive thematic maps. Candidate themes were judged by internal coherence, distinction, explanatory reach across cases and modes, and ability to accommodate contradictory material. The six themes were then organized processually as disruptions and absences, forms of relational disconnection, identity-protective meaning-making, and responses, rather than as six equivalent variables. Figure 1 summarizes this recursive development.

### 2.8. Longitudinal and Multimodal Analysis

Case-oriented analysis integrated each participant’s biography, relationships, transitions, living arrangements, intimacy, language of disconnection, distress, coping, diary materials, and changes across interviews. A longitudinal matrix compared the initial interview, diary/elicitation phase, and follow-up across ten dimensions: significant relationships; losses and transitions; social and emotional connection; intimacy and touch; usefulness and purpose; masculine identity; language of loneliness; psychological distress; coping and help-seeking; and future orientation. For each case, comparison asked what remained stable, what changed in language or relational circumstances, what evidence supported the participant’s interpretation, and what plausible alternative—including research elicitation—could explain change. Cross-case matrices then examined convergence and divergence by loneliness-label acceptance, living arrangement, relationship status, sexual orientation where relevant, health and mobility, caregiving, community participation, and material resources. The four reported trajectories were developed from recurring configurations of stability and change; they were non-exclusive interpretive patterns, not categories to which all 32 men were assigned. Their boundaries rested respectively on changed recognition, enacted relational change, continuity of guarded self-reliance, or compounding loss of relational opportunity. Contradictory cases were retained within the relevant pattern descriptions rather than forced into exclusive groups, and pattern frequencies are not reported because the purpose was explanatory comparison rather than classification. Visual and audio materials were interpreted through participants’ accounts and their spatial, material, sensory, and temporal contexts; loneliness was never inferred solely from empty rooms, solitary activities, or vocal characteristics.

### 2.9. Reflexivity and Methodological Rigor

Reflexive journals and the audit trail documented assumptions, emotional reactions, code development, candidate-theme revisions, and tensions between participant and researcher interpretations. Credibility was supported through prolonged and repeated engagement, triangulation across interviews and participant-generated materials, participant-led elicitation of diary meanings, case summaries, longitudinal matrices, contextualized quotations, and explicit comparison with negative and contrasting cases. These strategies were used for reflexive accountability and interpretive depth, not to claim a single reproducible coding truth or inter-coder consensus. The researcher actively tested alternatives to construing solitude, emotional restraint, activity, alcohol use, grief, role loss, or reluctance to seek help as unnamed loneliness. The initial sensitizing framework was retained only where accounts showed unwanted relational disconnection; chosen-solitude cases and stable forms of self-reliance established exclusions and limits.

### 2.10. Ethical Considerations

Institutional ethical approval was obtained, and the study followed the Declaration of Helsinki, Portuguese research-ethics standards, and the General Data Protection Regulation. Written consent was treated as ongoing and reconfirmed at each stage and before any use of participant-generated material. Participants could withdraw without penalty and request data deletion until four weeks after participation. Demographic and contextual details were generalized when necessary to reduce deductive identification, and no financial compensation was provided.

## 3. Results

Reflexive thematic analysis generated six interrelated themes: (1) loneliness without the word loneliness; (2) from usefulness to invisibility; (3) socially present but emotionally alone; (4) the masculine discipline of silence; (5) homes, routines, and objects marked by absence; and (6) reconstructing connection without surrendering autonomy. These themes operate at deliberately different points in a process rather than as six parallel variables: life-course and material disruptions altered valued roles and intimate routines; relational disconnection was experienced as invisibility, insufficient intimacy, or lack of consequentiality; identity-protective language and silence shaped recognition and disclosure; and autonomy-preserving action could sustain withdrawal or enable reconstruction. The themes did not represent mutually exclusive participant types. This processual organization distinguishes antecedents and contexts, forms of disconnection, meaning-making mechanisms, and responses.

The analytic pattern was not a simple opposition between lonely men and non-lonely men. Nineteen participants did not identify as lonely at baseline, yet several described prolonged silence, diminished purpose, absence of intimacy, or reluctance to seek contact. Conversely, men living alone who described solitude as chosen, satisfying, and consistent with their relational preferences functioned as theoretically decisive negative cases: they were not classified as experiencing unnamed loneliness. Other contrasts showed that co-residence or regular participation could coexist with unwanted emotional disconnection, while activity-based friendship could be sufficient when it matched the participant’s needs. Interpretive emphasis was therefore placed on the participant-described discrepancy between desired and experienced connection, the quality and reciprocity of relationships, the meanings of autonomy, and changes across the three study stages—not on living arrangement, mood, routine, or label rejection alone.

Across themes, direct quotations are used selectively to retain participants’ language while the accompanying analysis examines shared meanings, contradictions, contextual variation, and longitudinal change.

### 3.1. Contextual Contrasts Across the Maximum-Variation Sample

The deliberately heterogeneous sample clarified that the core processes did not map neatly onto demographic categories. Relationship status and living arrangement altered the form, not the simple presence, of disconnection: P03, widowed and living alone, became more willing to name loneliness at night, whereas P31 described emotional loneliness while co-residing with and caring for his wife; by contrast, men living alone who described solitude as chosen and satisfying provided negative cases. Mobility limitations sharpened the shift from contributor to recipient for P28, for whom dependence on his daughter constrained alternative ways of feeling useful, while accessible volunteering or neighborhood roles allowed other participants to retain reciprocity despite reduced capacity. Sexual orientation likewise did not determine a single trajectory: the gay, partnered participant quoted in Theme 4 drew on prior support and minority-community experience to reinterpret disclosure as strength, illustrating how available relational cultures could widen the meanings of masculinity and help-seeking. These contrasts are analytic illustrations rather than subgroup comparisons and should not be read as prevalence claims.

### 3.2. Theme 1: Loneliness Without the Word Loneliness

The first theme concerned a recurrent discrepancy between lived descriptions of disconnection and willingness to accept the word loneliness. Participants often treated loneliness as a categorical and extreme condition belonging to a person who had “nobody at all.” The existence of children, neighbors, acquaintances, or occasional telephone contact was therefore used as evidence that the category did not apply, even when those relationships did not interrupt long periods of silence or provide emotional safety. This threshold protected participants from an identity associated with abandonment, incapacity, or social failure, but it also narrowed what could be recognized as a legitimate relational need.

“*It is not loneliness. Loneliness sounds dramatic. It is more that nothing is waiting for me. I am just no longer needed. I wake up, make coffee, and there is no reason to hurry.*”

(P19, aged 69, retired and living alone)

“Nothing waiting for me” condensed several analytic dimensions: absence of anticipation, responsibility, reciprocal expectation, and a social role requiring the participant’s presence. His account did not primarily describe insufficient contact; it described a day without relational consequence. Similar linguistic substitutions included boredom, emptiness, having too much time, being forgotten, and not having anyone with whom to talk “properly.” These formulations made distress more compatible with masculine self-containment because they located the problem in time, routine, or circumstance rather than explicitly in emotional need.

Participants also differentiated superficial exchange from being known. Men could report frequent encounters in cafés, shops, community activities, or shared households while describing no person to whom fear, grief, or uncertainty could be disclosed. The relevant deficit was therefore not the absence of speech but the absence of a relationship in which speech could carry personal risk. This distinction explains why objective contact and subjective connection sometimes diverged sharply.

“*At the first interview, I said I was not lonely. I think I did not want that word attached to me. Now I would say there are moments when I am lonely, especially at night. Admitting it does not mean I cannot manage my life.*”

(P03, aged 72, widowed)

This longitudinal shift represented increasing interpretive flexibility rather than a simple increase in symptoms. Naming loneliness became possible once the participant separated it from incompetence and dependency. Other men retained their rejection of the term, and in some cases that rejection was consistent with valued solitude rather than denial. The theme therefore concerns the social and identity conditions under which loneliness can be named, not an assumption that every indirect expression conceals an unacknowledged condition.

### 3.3. Theme 2: From Usefulness to Invisibility

A second theme linked connection to usefulness, contribution, and recognition. Work, caregiving, practical competence, and family responsibility had organized many participants’ identities and relationships. Retirement or declining health could remove not only activity but also the experience of being consulted, expected, and able to affect other people’s lives. Loneliness consequently appeared as a loss of social position: participants remained physically present but no longer occupied a role through which their presence was consequential.

“*At work, people came to me because I knew how things were done. After retirement, the telephone stopped. It was as if the person they needed had disappeared, although I was still here.*”

(P14, aged 68, retired civil servant)

The contrast between being “still here” and having “disappeared” showed that invisibility was relational rather than literal. What had vanished was a recognized identity built through expertise and reciprocal dependence. Men who had anticipated retirement as freedom sometimes found that unrestricted choice had little meaning without responsibility or shared purpose. This finding also qualified accounts that equate autonomy with well-being: autonomy was valued, but autonomy without recognition could become emptiness.

Family change produced a parallel dynamic. Participants were proud of adult children’s independence yet missed ordinary requests to repair, transport, advise, or care. Digital services and professionalized support sometimes displaced practical exchanges that had functioned as expressions of affection. Because several men communicated care primarily through doing rather than verbal disclosure, the loss of practical usefulness also removed a culturally acceptable route to intimacy.

“*I was always the one who carried things, drove people, took care of problems. Now my daughter has to take me to appointments. She does it willingly, but I feel I am becoming another obligation in her week.*”

(P28, aged 81, widowed)

The transition from giving to receiving care exposed a tension between reciprocity and burdensomeness. Participants sometimes reduced contact to protect children from obligation, but this self-restriction also protected them from possible refusal and deepened disconnection. The pattern was strongest when health and mobility limitations reduced alternative sources of contribution. Usefulness was therefore not merely a traditional masculine ideal; it operated as a relational currency through which participants experienced dignity, belonging, and permission to remain close to others.

### 3.4. Theme 3: Socially Present but Emotionally Alone

The third theme showed that co-residence, partnership, friendship, and community participation did not necessarily provide emotional intimacy. Some long-term relationships remained loyal and practically cooperative yet offered little language for vulnerability. Illness and caregiving could further transform intimacy into coordination, concentrating conversation on medication, appointments, shopping, and household management. These relationships were not described as devoid of love; rather, emotional loneliness emerged within relationships whose practical functions remained intact.

“*We talk about medication, shopping, bills, and appointments. We manage the house very well. But sometimes I think we have forgotten how to ask each other how we are.*”

(P31, aged 78, caregiver for his wife)

The phrase “manage the house” positioned the couple as an effective unit while simultaneously revealing an absence of mutual emotional inquiry. Among participants caring for partners with cognitive or physical decline, loneliness could involve the gradual loss of the person who had known their history, even while that person remained physically present. Co-residence could therefore intensify rather than resolve loneliness by making relational change continuously visible.

Friendships often provided loyalty, humor, shared activity, and practical reliability but limited emotional disclosure. For some men this form of companionship was sufficient and should not be judged against a disclosure-centered ideal. For others, the inability to move from “speaking” about football, politics, or practical problems to “talking” about the self became painful during bereavement, illness, or retirement. The distinction depended on whether activity-based connection matched the participant’s needs at that moment.

“*People assume that at my age you miss conversation. Of course I miss conversation. But I also miss someone putting a hand on my shoulder without it being a doctor.*”

(P26, aged 79, widowed)

This account located loneliness in the body and distinguished affectionate touch from clinical contact. Widowed, divorced, and unpartnered participants described missing shared sleep, spontaneous touch, bodily familiarity, and being desired, yet they frequently anticipated embarrassment or ageist judgment if they disclosed sexual loss. The findings therefore broaden emotional loneliness to include physical and erotic recognition. Social presence could not compensate for the absence of a specific kind of intimacy, and grief was culturally easier to acknowledge when framed as missing companionship or domestic routine than when framed as missing a partner’s body.

### 3.5. Theme 4: The Masculine Discipline of Silence

Participants described emotional silence as a learned discipline rather than a simple inability to identify feelings. Many had been socialized to continue working, solve practical problems, remain composed, and avoid burdening others. These practices had often been adaptive during hardship and were associated with dignity and reliability. Their costs became more visible when work, partnership, or physical capacity no longer supplied a structure through which distress could be contained.

“*I would not say depressed. I become irritated. Everything annoys me. Then I stay at home because I know I am not good company.*”

(P12, aged 69, single)

Irritability and withdrawal formed a self-reinforcing sequence. Staying home was presented as protection of others and preservation of control, yet it reduced opportunities for corrective contact and made the participant’s deteriorating mood less visible. Similar substitutions included poor sleep, bodily tension, lack of motivation, alcohol use, or the conventional response “I am fine.” The analysis suggests that silence was actively maintained through judgments about what others wished to hear, what counted as a legitimate problem, and what disclosure might release.

Help-seeking became more acceptable when framed as practical, bounded, or oriented toward a specific solution. Interventions explicitly organized around loneliness or unrestricted emotional talk were often viewed as passive, stigmatizing, or incompatible with self-respect. This was not universal: prior psychotherapy, caregiving, support-group participation, and minority-community networks had enabled some men to reinterpret disclosure as competence rather than weakness.

“*My generation confused strength with silence. Sometimes the stronger thing is to tell another person that you are not coping.*”

(P10, aged 70, gay and partnered)

This counter-narrative demonstrates that masculinity functioned as a contested repertoire rather than a fixed barrier. Participants could mobilize masculine values in opposite directions: endurance justified silence, while courage and responsibility justified disclosure. Clinically, the relevant question is therefore not whether a man conforms to masculinity in the abstract, but which meanings of strength, control, reciprocity, and responsibility are available in a particular relational context.

### 3.6. Theme 5: Homes, Routines, and Objects Marked by Absence

Participant-generated materials made visible the spatial, sensory, and temporal organization of loneliness. Empty chairs, unused rooms, single place settings, tools, clothing, background television, and recordings made at difficult times of day were not treated as self-evident symbols. Their significance emerged through the participant’s explanation and through comparison with earlier and later accounts. The home appeared as an active repository of relationships: objects preserved continuity while also repeatedly staging absence.

“*We always sat there after dinner. I still use my chair, but I have never moved hers. It would feel like admitting she is not coming back.*”

(P03, aged 72, widowed)

The second chair simultaneously maintained a bond, protected the participant from a final symbolic separation, and materialized the impossibility of restoring the former routine. Its meaning was therefore ambivalent rather than simply pathological. Comparable objects, including work tools and clothing, connected participants to earlier identities as partners, workers, caregivers, or problem-solvers. Their continued presence could comfort, but their non-use could also confirm a loss of relational purpose.

“*At six o’clock, the building changes. You hear families arriving, doors opening, children. My apartment becomes quieter because I hear everyone else beginning their evening.*”

(P15, aged 71, divorced)

This audio-diary account showed that loneliness was intensified through contrast. The participant’s apartment did not literally become quieter; neighboring sounds made his lack of shared evening routine more perceptible. Meals, Sundays, returning home, and the transition from daylight to evening similarly concentrated awareness of absence. These temporal patterns were less evident in initial interviews and illustrate the added value of diaries for identifying experiences that are repetitive, sensory, or difficult to summarize retrospectively.

Routine had a dual function. Walking to a café, caring for an animal, gardening, or buying a newspaper supported continuity and weak-tie recognition. Conversely, rigid busyness and constant television could prevent silence from becoming conscious without creating meaningful contact. The distinction was not the behavior itself but whether it generated reciprocity and connection or primarily narrowed the space in which distress could be felt.

### 3.7. Theme 6: Reconstructing Connection Without Surrendering Autonomy

The final theme concerned how participants rebuilt connection while protecting dignity, agency, and control. Men were more receptive to opportunities organized around contribution, skill, shared activity, or a clear social purpose than to initiatives that defined them primarily as vulnerable recipients. This preference was not merely a matter of program branding. It reflected a desire to enter relationships as someone able to give as well as receive.

“*I would never go to a group called ‘lonely men.’ That would feel like accepting a label. But if it were a group to repair things or help the neighborhood, I would consider it.*”

(P17, aged 70, divorced)

The hypothetical repair group preserved a valued identity and created reciprocity from the outset. Volunteering had a similar effect when another person expected the participant to arrive and depended on his contribution. Belonging was repeatedly described through expectation: connection meant not only having access to others but having a recognized place that could not be filled indifferently.

Several men also identified protective interpersonal patterns during follow-up. Waiting for children or friends to initiate contact reduced the immediate risk of rejection but converted silence into evidence that one did not matter. Small changes—calling first, accepting invitations, recontacting old friends, joining walking or cultural groups, or speaking more honestly with family—were meaningful when they altered this passive test of significance.

“*I realized I was testing people without telling them. I waited to see who would call, and when nobody called, I decided I did not matter. Now I call first sometimes.*”

(P05, aged 73, divorced)

The participant did not relinquish independence; he revised it from self-sufficiency to relational agency. Across accounts, sustainable support respected choice over when, how, and by whom help was provided. This relational form of autonomy allowed participants to remain decision-makers while acknowledging interdependence. Connection was therefore reconstructed most effectively when support increased participation and reciprocity rather than replacing agency.

### 3.8. Longitudinal Patterns of Change

Comparison across the three analytic phases identified four broad patterns: increasing recognition, active reconstruction, stable guarded self-reliance, and accumulating disconnection. They summarize recurrent temporal mechanisms rather than divide the sample into four groups: recognition concerns a changed interpretive frame; reconstruction requires enacted relational change; guarded self-reliance denotes continuity, whether satisfying or ambivalent; and accumulating disconnection reflects compounding losses in both relationships and practical opportunity. Participants could show more than one pattern, and discordant material was retained rather than resolved by forced assignment. The theoretical value of the formulation lies in separating change in naming from change in distress, and personal agency from the material conditions that enable connection.

#### 3.8.1. Increasing Recognition

Some men gradually differentiated chosen solitude from unwanted disconnection and acknowledged that contact did not necessarily provide intimacy. Diary and elicitation work often supported this shift by making repetitive silence, absent relationships, and difficult times of day visible. Greater recognition did not invariably mean greater distress; in several cases it reduced shame by making loneliness compatible with competence.

#### 3.8.2. Active Reconstruction

A second group initiated contact, accepted invitations, joined activities, volunteered, or negotiated support differently. Improvement was associated less with a larger number of contacts than with reciprocity, emotional relevance, and being expected. The men who changed most visibly had opportunities that matched their identities and practical circumstances.

#### 3.8.3. Stable Guarded Self-Reliance

Some participants consistently valued limited contact and did not seek change. For several, this represented satisfying solitude and should not be reclassified as pathology. Others displayed unresolved ambivalence: they described emptiness or lack of intimacy while rejecting support. The distinction could not be made from living arrangements or language alone and required attention to affect, context, contradiction, and change over time.

#### 3.8.4. Accumulating Disconnection

A final pattern involved worsening disconnection after health deterioration, bereavement, caregiving strain, reduced mobility, or declining family contact. In these cases, previously effective coping practices depended on physical, financial, and environmental resources that were no longer available.

“*Before, I could always go out when the house became too quiet. Now my legs do not allow it. The loneliness has not changed, but my ways of escaping it have disappeared.*”

(P25, aged 83, widowed)

This trajectory qualified individualistic explanations of loneliness. Motivation and willingness to connect were insufficient when mobility, transport, accessible environments, financial resources, or suitable community opportunities were absent. Longitudinal change therefore reflected both personal interpretation and material opportunity.

### 3.9. Integrative Interpretation: Unnamed Loneliness as a Relational and Identity-Protective Process

Across the six themes, unnamed loneliness involved more than avoiding a word. The four candidate processes were present in the sensitizing framework before data collection and are not claimed as discoveries of this analysis. Empirical analysis changed their status and scope in three ways: it made unwanted relational discrepancy the threshold for inclusion; showed that the processes could be sequential, overlapping, absent, or reversible rather than a fixed typology; and located them within a broader process linking life-course disruption, relational consequentiality, identity protection, and either withdrawal or reconstruction. The data also contradicted any assumption that rejection of the label necessarily indicated nonrecognition, because some participants described satisfying solitude.

First, nonrecognition occurred when participants did not interpret their experience as loneliness because they retained some contact, lived with another person, or equated loneliness with total abandonment. Second, linguistic substitution translated relational distress into culturally safer terms such as boredom, irritability, emptiness, excessive time, or loss of purpose. These processes shaped what participants could recognize in themselves and what clinicians or relatives might hear.

Third, identity protection distanced participants from the stigmatized image of the dependent, socially unsuccessful, or emotionally uncontrolled older man. Fourth, relational concealment occurred when distress was recognized but withheld to avoid burdening others, threatening reciprocity, or risking rejection. Concealment could therefore be both caring and self-protective, while unintentionally reducing the likelihood that others would respond.

The processes were neither universal nor inevitably maladaptive. Self-reliance could sustain dignity, chosen solitude could be meaningful, activity-based friendships could be sufficient, and silence could preserve functioning. They became problematic when they prevented participants from obtaining the quality of connection they themselves desired or when structural constraints removed alternative routes to participation.

The findings consequently position loneliness not simply as the absence of people but as a threatened relationship between self and social world: not being known, expected, useful, touched, desired, or able to contribute without surrendering autonomy. This interpretation integrates emotional, social, existential, embodied, and identity-related dimensions while retaining the diversity of older men’s experiences.

## 4. Discussion

This qualitative longitudinal study explored how 32 community-dwelling older men in Lisbon experienced and communicated loneliness, relational disconnection, and associated psychological distress. Six interrelated themes were developed: loneliness without the word loneliness; the transition from usefulness to invisibility; being socially present but emotionally alone; the masculine discipline of silence; homes, routines, and objects marked by absence; and reconstructing connection without surrendering autonomy. The longitudinal analysis further suggested four broad patterns of change: increasing recognition, active reconstruction, stable guarded self-reliance, and accumulating disconnection.

Taken together, the findings extend conceptualizations of loneliness beyond objective isolation, limited network size, or the explicit statement “I am lonely.” For many participants, loneliness was embedded in perceived uselessness, insufficient emotional intimacy, disruption of valued roles, absence of reciprocal recognition, and the belief that their presence no longer mattered to others. The findings therefore support a relational and identity-sensitive understanding of loneliness in which the central issue is not simply whether other people are present, but whether a man experiences himself as known, valued, expected, needed, and able to communicate vulnerability within his relationships.

The study’s contribution is not the de novo discovery of four processes that were already specified provisionally. It is the empirical refinement of unnamed loneliness as a bounded, relational, and potentially reversible sensitizing concept, together with two more specific advances. First, the accounts distinguished social presence from relational consequentiality: connection involved being expected, needed, consulted, emotionally known, and able to contribute, not simply encountering other people. Second, longitudinal change in loneliness self-identification could reflect a changed interpretation of relational need rather than increased symptom severity. Nonrecognition, linguistic substitution, identity protection, and relational concealment help explain how these processes become more or less speakable, but they should not be interpreted as deficits in emotional competence or imposed where participants describe chosen, satisfying solitude.

Relational consequentiality is not proposed as an entirely new construct or as a replacement for mattering, purpose, usefulness, reciprocity, social role, or generativity. It is closest to relational mattering—the sense of being important, attended to, and depended upon—which has particular relevance to the well-being of older adults (Flett & Heisel, 2021). The present formulation nevertheless gives that broad perception a more situated and processual focus: it asks how a man’s presence makes a recognizable difference within particular relationships through being expected, consulted, emotionally known, relied upon, or able to contribute.

The neighboring constructs illuminate different parts of this pattern. Purpose concerns direction and meaning and may be pursued outside close relationships; usefulness concerns perceived capacity, which becomes relationally consequential only when it is recognized or enacted with others; reciprocity concerns mutual exchange, whereas consequentiality can also be experienced through attention, consultation, or emotional recognition when exchange is temporarily asymmetrical. A social role names a formal or informal position, while consequentiality describes the lived relational effect of occupying—or losing—that position. Generativity emphasizes concern and action directed toward sustaining others and future generations (McAdams & de St. Aubin, 1992), whereas relational consequentiality also includes horizontal ties, receiving recognition, and the immediate sense that one’s presence matters. Its added value here is therefore integrative and context-specific: it connects these adjacent ideas to later-life loneliness among men and explains why contact alone may not restore belonging after retirement, widowhood, disability, caregiving change, or shifts in masculine identity.

### 4.1. Loneliness Without Explicit Self-Identification

The finding that some participants described prolonged silence, emptiness, lack of meaningful conversation, or absence of social expectation while rejecting the term loneliness is consistent with evidence that older men may be less likely than women to identify themselves directly as lonely after levels of loneliness measured indirectly are taken into account. Ratcliffe et al. (2024), for example, found that older men were less likely to endorse a direct loneliness question than older women with comparable loneliness-scale scores. This does not necessarily mean that men experience a fundamentally different form of loneliness. It suggests that willingness to adopt the label may be shaped by gendered meanings, stigma, and assumptions about what qualifies someone as lonely.

Participants frequently reserved the loneliness label for an imagined person who had “nobody,” thereby excluding themselves because they had children, neighbors, acquaintances, or occasional interactions. This categorical understanding obscured the distinction between the existence of relationships and their emotional quality. A participant could therefore report having people in his life while simultaneously describing entire days without conversation or an inability to speak honestly with anyone.

The distinction participants made between “speaking” and “talking” is especially important. Routine interaction may provide social stimulation and recognition but may not meet needs for emotional disclosure, attachment, validation, or intimacy. This supports multidimensional models that distinguish social loneliness from emotional and existential loneliness. It also demonstrates why assessments based solely on living arrangements, frequency of contact, or network size can fail to identify meaningful relational deprivation.

The findings are consistent with Willis and Vickery’s (2022) study of older men living alone in England, in which maintaining silence around loneliness and distress was a central theme. Their participants described “getting on with it,” becoming stuck in loneliness, and using temporary distractions within the home. The present study extends this work by suggesting that silence may occur not only among men who live alone but also among partnered men and those with apparently active social lives.

It is nevertheless important not to treat rejection of the loneliness label as proof of denial. Some men genuinely valued solitude and experienced limited social contact as compatible with autonomy, psychological comfort, and meaningful living. The concept of unnamed loneliness is therefore most useful when there is evidence of unwanted disconnection, rather than when a researcher merely observes that a participant spends substantial time alone. The distinction must remain grounded in the person’s broader narrative, including whether solitude is chosen, satisfying, sustainable, and consistent with his relational needs.

The longitudinal findings add further nuance. Some participants became more willing to name loneliness during follow-up without necessarily becoming more distressed. For these men, recognizing loneliness appeared to reduce the incompatibility between relational need and masculine autonomy. The admission “I sometimes feel lonely” could be incorporated into a self-concept that still emphasized competence and independence. This suggests that naming loneliness may be an interpretive development rather than simply an indicator of symptom severity.

### 4.2. Usefulness, Recognition, and the Social Meaning of Older Manhood

The second major finding concerned the close relationship between social connection and feeling useful. Participants frequently described retirement, declining health, reduced family dependence, and loss of occupational responsibility as transitions from social visibility to perceived redundancy. Their distress was not limited to having less to do. It involved the loss of roles through which they had experienced competence, recognition, and reciprocal value.

This finding is consistent with evidence that purpose in life is closely connected to loneliness and well-being among older men. Research with men aged 60 and older has indicated that purpose may protect against loneliness by providing direction, structure, and a continuing sense of contribution. Longitudinal evidence also identifies employment, volunteering, partnership, health, social participation, and neighborhood belonging as important correlates or predictors of men’s loneliness across the life course.

For many participants, work had been a relational institution as well as an economic activity. Employment provided routines, shared problems, recognition of expertise, and evidence that others depended on them. Retirement disrupted this structure. The participant who described his telephone ceasing to ring after retirement conveyed a loss of interpersonal demand: he remained physically present but no longer occupied the same position in other people’s social worlds.

This interpretation is compatible with critical masculinities scholarship demonstrating that masculine identity in later life is often negotiated through autonomy, productivity, responsibility, and practical contribution. These values are not inherently harmful. They may sustain resilience, perseverance, and commitment to others. However, vulnerability can arise when a man’s sense of worth depends too narrowly on capacities that are threatened by retirement, illness, disability, or age-related changes. Older men may then experience declining usefulness not merely as a practical change but as a challenge to identity and belonging.

The findings also complicate individualistic understandings of loneliness. Several men attempted to manage disconnection through greater personal activity, but the deeper problem was not always a lack of hobbies. It was the absence of reciprocity and social expectation. Being busy did not necessarily reproduce the experience of mattering to someone else. The difference between merely attending an activity and being expected to arrive was therefore psychologically significant.

This distinction has practical implications. Interventions that offer older men passive participation or generic social contact may be less meaningful than opportunities that confer responsibility, competence, and reciprocal contribution. Volunteering, mentoring, repairing, teaching, caregiving, organizing activities, and contributing to neighborhood projects may provide stronger pathways to connection because they address both relational need and identity. Community activities organized around practical contribution can engage masculine repertoires constructively rather than requiring men to abandon them. Evidence concerning Men’s Sheds and related community initiatives suggests that activity-based environments can facilitate connection by combining practical engagement, informal conversation, and contribution.

The participants’ fear of becoming burdensome was equally important. Some men restricted contact with adult children because they did not want to become an additional obligation. Although framed as care for family members, this restraint also protected participants from potential rejection and concealed their relational needs. The result was a self-reinforcing process: men avoided calling because they feared burdening others, relatives interpreted the absence of contact as independence, and unmet need remained invisible.

This process may have particular clinical importance where perceived burdensomeness, loss of autonomy, and diminished purpose coexist with hopelessness or thoughts about death. Qualitative research with men over 80 has shown that control, independence, loneliness, residential dependency, and not wanting to burden others can become intertwined with assessments of whether life remains worth living. The present findings reinforce the need for clinicians to explore what phrases such as “I do not want to be a burden” mean within an older man’s life, rather than regarding them as ordinary expressions of modesty or independence.

### 4.3. Social Contact Without Emotional Intimacy

The finding that men could be socially present yet emotionally alone challenges the assumption that co-residence, partnership, or community participation necessarily protects against loneliness. Several participants had regular contact with spouses, family members, friends, or neighbors but lacked a relationship in which they felt able to communicate psychological distress.

Partnered participants sometimes described relationships characterized by loyalty, practical cooperation, and shared history but limited emotional disclosure. This does not mean that such relationships were deficient or inauthentic. Practical care and companionship were often highly valued. However, the findings indicate that relational presence and emotional availability should not be conflated.

The importance of partnership may be especially pronounced among men whose spouse serves as their primary or only source of emotional disclosure and social coordination. Research indicates that men’s social isolation is strongly shaped by marital and partnership history, with never-married men and those who have experienced relationship disruption often facing greater vulnerability. Older men’s loneliness has also been associated with partnership status, while men may rely more heavily than women on a partner as a principal source of intimacy and support.

The experiences of men caring for partners with dementia or serious illness further demonstrate that physical proximity cannot be used as a proxy for reciprocal connection. Caregiving could provide responsibility, commitment, and purpose while also involving the gradual loss of conversation, recognition, shared decision-making, sexual intimacy, and emotional reciprocity. Previous research has documented the profound effects of dementia on couple relationships, including changes in emotional closeness, identity, intimacy, and sexuality.

Participants’ descriptions of male friendships similarly revealed the distinction between practical reliability and emotional accessibility. Activity-based friendships were often meaningful and should not be evaluated against an idealized model in which emotional disclosure is the only marker of closeness. Shared humor, practical assistance, routine presence, and companionship may constitute culturally and personally meaningful intimacy. However, some participants explicitly wanted to communicate distress but did not know how to initiate such conversations or how their friends would respond.

This pattern aligns with research indicating that men’s social connectedness can be supported through shared activity while emotional restriction and uncertainty about responding to disclosure can limit the depth of support available. Masculine norms do not simply reduce the number of relationships; they may shape what can be communicated within those relationships.

The findings therefore suggest that interventions should not focus exclusively on enlarging social networks. Increasing the number of contacts may provide limited benefit when men continue to lack relational safety, reciprocity, or emotional recognition. Programs should also help men deepen selected existing relationships, develop confidence in initiating personal conversations, and respond constructively when another man discloses distress.

### 4.4. The Masculine Discipline of Silence

The theme of masculine silence demonstrated that non-disclosure was not simply an individual communication deficit. Participants described emotional restraint as a learned discipline associated with strength, responsibility, control, and protection of others. Many had grown up in contexts where distress was managed through work, endurance, distraction, or silence.

These findings support research showing that masculine norms emphasizing self-reliance, emotional control, stoicism, and invulnerability can constrain help-seeking and social connection. However, the relationship is contextual rather than deterministic. Men may adopt, resist, or reinterpret these norms depending on age, relationships, health, social class, sexuality, and previous experiences of support.

Silence sometimes allowed participants to preserve everyday functioning. Continuing with work, routine, or practical tasks could prevent emotional overwhelm and support resilience during bereavement or illness. It would therefore be reductive to classify all emotional restraint as maladaptive. Difficulties arose when silence became the only acceptable response and prevented men from obtaining support even when established coping practices were no longer sufficient.

The phrase “I am fine” illustrated this tension. It functioned simultaneously as social convention, identity performance, and a boundary against further inquiry. Some participants assumed that everyday questions about well-being were not genuine invitations to disclose. Consequently, others may have interpreted socially expected reassurance as evidence that no support was needed.

This has implications for primary care and community services. Asking a single general question such as “Are you okay?” may be insufficient. More concrete, contextual questions may be better able to elicit distress, such as: “Who do you speak to when something is worrying you?” “What happens on the days when you do not feel like leaving home?” “Who would notice if you were having a difficult week?” “Do you sometimes avoid contacting people because you do not want to burden them?” “Since retirement or bereavement, when do you feel least useful?” “Are there times when life feels empty or without purpose?”

These questions do not presume that the man is lonely, but they create opportunities to discuss relational need through everyday experience.

Participants were often more accepting of support when it was framed as practical, purposeful, and time-limited. This pattern is consistent with contextual models of men’s help-seeking, which emphasize that men’s engagement depends on how a problem and its proposed solution are framed. Men may avoid services perceived as stigmatizing, passive, or threatening to autonomy while engaging with support that emphasizes collaboration, competence, action, and personal agency.

The findings also demonstrate possibilities for change. Some participants explicitly challenged the equation of strength with silence and reframed disclosure as responsibility, courage, or maturity. Such accounts resist deficit narratives that portray older men as uniformly emotionally restricted. They suggest that gender-sensitive practice should not attempt to eliminate masculinity but should expand the forms of masculinity available to men. Care, interdependence, emotional honesty, and help-seeking can be presented as compatible with dignity, autonomy, and responsibility.

### 4.5. Loneliness as Spatial, Material, Temporal, and Embodied

The participant-generated diaries and elicitation interviews revealed dimensions of loneliness that may have remained less visible in conventional interviews. Homes, objects, sounds, routines, meals, and times of day carried relational meaning. Empty chairs, unused tools, single place settings, and background television were not inherently symbols of loneliness; their significance arose from the participants’ narratives concerning absence, memory, identity, and changed routines.

This finding reinforces the value of visual and elicitation methods for studying experiences that are difficult to communicate directly. Loneliness was not experienced as a continuous, context-free state. It often intensified in particular places and at specific times, including during meals, evenings, weekends, or upon returning to an empty home.

For widowed participants, domestic objects provided continuing bonds with deceased partners. The home could simultaneously preserve relational history and repeatedly make absence perceptible. This supports earlier qualitative research showing that loneliness after widowhood is deeply connected to everyday spaces and the visible absence of a spouse from familiar routines.

The findings also demonstrate that loneliness is embodied. Participants described missing touch, shared sleep, physical affection, and the experience of being desired. These needs were not adequately represented by measures of social contact. A man could speak with relatives daily yet experience an absence of physical intimacy that remained difficult to communicate because sexuality in later life was stigmatized or treated as irrelevant.

Research on later-life sexuality consistently challenges assumptions that older adults become asexual or cease to value intimacy. Qualitative studies have found that sexuality and intimacy remain important but are shaped by health, partner availability, cultural expectations, body image, privacy, and ageism. Older adults may also encounter barriers when seeking sexual-health advice because both patients and health professionals hesitate to initiate these conversations.

The participants’ distinction between missing conversation and missing touch indicates that loneliness interventions should not reduce intimacy to social participation. Clinical and community assessments should create respectful opportunities to discuss affection, partnership, sexuality, and bodily connection without assuming heterosexuality, partnership, or sexual activity.

### 4.6. Reconstructing Connection While Preserving Autonomy

The final theme showed that older men were not passive recipients of loneliness. Participants adopted multiple strategies to maintain or rebuild connection, including volunteering, joining community activities, contacting friends, caring for animals, sustaining routines, and redefining independence.

The most acceptable forms of connection combined agency with reciprocity. Participants wanted to choose how they engage, contribute something of value, and avoid being positioned solely as vulnerable service users. A group explicitly advertised for “lonely men” was likely to be rejected because participation would require accepting a stigmatized identity. The same participants might engage in a workshop, repair project, neighborhood initiative, walking group, educational activity, or volunteering program that indirectly facilitated connection.

This finding is consistent with the previous Portuguese qualitative study of older men’s social connectedness, which identified family relationships, friendships, community participation, health, mobility, and personal initiative as important dimensions of connection. The present study adds that participation may be especially meaningful when it enables men to experience themselves as expected, competent, and useful.

Reconstructed connection was not limited to formal programs. Small interpersonal changes, such as initiating a telephone call or accepting an invitation, could interrupt patterns of protective withdrawal. One participant’s realization that he had been “testing” others by waiting for them to make contact illustrates how loneliness may become self-maintaining. Avoiding initiation protects against rejection but can also produce the very absence of contact that appears to confirm one’s insignificance.

This interpretation should not lead to blaming lonely men for their circumstances. The ability to initiate and sustain connection depends on health, mobility, income, transport, accessible public spaces, digital literacy, and the availability of appropriate social opportunities. The pattern of accumulating disconnection showed that motivation alone was insufficient when physical or environmental resources deteriorated.

The World Health Organization’s social-connection framework emphasizes that loneliness is influenced by individual, relational, community, and structural conditions. Its recommendations include strengthening community infrastructure, transportation, accessible services, and policies addressing marginalization and discrimination. The present findings support this multilevel approach: loneliness cannot be addressed adequately by instructing individuals to “socialize more” without ensuring that they have accessible, meaningful, and socially acceptable opportunities to do so.

### 4.7. A Conceptual Model of Unnamed Loneliness

The findings suggest that unnamed loneliness can be understood as a relational and identity-protective process rather than as a hidden condition that researchers simply need to expose.

The process may begin with a mismatch between existing relationships and desired forms of connection. This mismatch may involve insufficient intimacy, loss of a valued role, lack of reciprocal recognition, or absence of purpose. However, the experience may not be interpreted as loneliness because the man retains some social contact, does not live alone, or associates loneliness exclusively with abandonment.

The experience may then be communicated through linguistic substitution. Rather than speaking of loneliness, a man may describe boredom, irritability, emptiness, uselessness, excessive time, loss of routine, or the belief that nobody needs him. These alternative descriptions are not merely euphemisms; they may reflect the way distress is genuinely experienced and organized.

Identity protection occurs when rejecting the loneliness label helps preserve dignity, competence, and continuity with a self-concept based on independence. The label may threaten to reposition the man from someone who manages his life to someone perceived as socially unsuccessful or dependent.

Finally, relational concealment occurs when a man recognizes his need but withholds it to protect others, avoid shame, retain control, or prevent rejection. Silence then becomes both protective and isolating.

These processes may be moderated by life-course transitions, health, relationship history, socioeconomic circumstances, sexual orientation, community context, and access to acceptable forms of support. They are also potentially reversible. Participants’ longitudinal accounts showed that recognition, disclosure, and connection could change when relational needs were reframed as compatible with autonomy and when opportunities for meaningful contribution became available.

The concept should not be reified prematurely as a fixed typology or diagnostic entity. Its value lies in sensitizing researchers and practitioners to indirect expressions of relational distress. Future empirical research should determine whether the processes identified here are evident across other groups of men and cultural contexts.

### 4.8. Implications for Clinical Practice

The findings have several implications for psychologists, primary-care professionals, geriatric services, and community practitioners.

First, loneliness should not be assessed only by asking whether a patient feels lonely. Direct questions remain valuable, but they should be supplemented with exploration of relationships, roles, routines, intimacy, and perceived usefulness. Clinicians should attend to expressions such as “I have no reason to get up,” “I do not want to bother anyone,” “the days are all the same,” or “nobody needs me anymore.”

Second, the existence of relatives or a co-resident partner should not be assumed to indicate adequate emotional support. Assessment should examine whether the person has someone with whom he can communicate honestly and whether existing relationships provide reciprocity, recognition, and intimacy.

Third, professionals should explore retirement, bereavement, disability, and caregiving as identity transitions rather than only as stressful events. Questions about what the man has lost the ability to contribute may reveal important sources of distress.

Fourth, mental health support may be more acceptable when presented collaboratively and linked to concrete goals. Gender-sensitive practice can preserve agency by inviting men to identify problems, strengths, and preferred forms of support rather than positioning them as passive recipients of care.

Fifth, comments concerning burdensomeness, loss of purpose, or the belief that one’s absence would not matter warrant careful assessment, particularly when accompanied by withdrawal, alcohol misuse, deteriorating health, or hopelessness. Older men may communicate suicidality indirectly, and emotional restriction has been associated with barriers to disclosure and elevated risk.

Finally, conversations about intimacy should include emotional, physical, romantic, and sexual dimensions. Professionals should not assume that sexuality is irrelevant because of age, widowhood, disability, or chronic illness.

### 4.9. Implications for Community Intervention and Policy

The findings support community approaches that are age-friendly, gender-sensitive, and contribution-oriented. Programs should avoid defining participants solely through deficits such as loneliness, frailty, or dependency. More acceptable entry points may include learning, mentoring, volunteering, practical projects, walking, cultural participation, caregiving support, and intergenerational activities.

Programs should provide opportunities for both activity and conversation. Shared tasks may create an environment in which trust develops gradually, and disclosure becomes possible without being demanded. Facilitators should be trained to recognize indirect distress and respond appropriately without converting informal community spaces into unwanted clinical settings.

Municipal and national policies should also address structural barriers. Accessible transportation, affordable activities, safe public spaces, neighborhood facilities, and support for men with reduced mobility can determine whether social participation is realistically possible. Lisbon’s urban density may provide opportunities for contact, but physical proximity does not ensure accessibility or belonging.

Intervention strategies should additionally recognize diversity among older men. Gay and bisexual men, migrants, men without children, men with disabilities, caregivers, economically disadvantaged men, and men with disrupted relationship histories may encounter distinct barriers. Sexual-minority older adults may rely strongly on chosen family and friendship networks while also experiencing ageism, minority stress, bereavement, or concerns about discrimination in health and social-care settings. Loneliness has also been identified as a potentially important mechanism linking sexual-minority status with depressive symptoms in later life.

### 4.10. Strengths

A principal strength was the qualitative longitudinal, multimethod design. Repeated engagement allowed the researcher to examine not only how participants described loneliness but also how those descriptions changed over time.

The use of participant-generated photographs, audio recordings, written notes, and objects broadened the forms of knowledge available to the study. These methods revealed spatial, temporal, sensory, and embodied aspects of loneliness that may have remained less visible in a single verbal interview.

The recruitment strategy did not require men to identify as lonely. This enabled the study to explore experiences among participants who rejected or did not use the term, directly addressing the central concept of unnamed loneliness.

Maximum-variation sampling also supported examination of different living arrangements, relationship histories, health conditions, and social contexts. Case-oriented longitudinal analysis preserved the coherence of individual biographies, while cross-case analysis identified shared patterns and meaningful variation.

Finally, the reflexive analytic approach reduced the risk of treating the researcher’s initial assumptions as self-evident. The study explicitly considered alternative explanations and did not automatically classify solitude, self-reliance, practical coping, or limited emotional disclosure as pathology.

### 4.11. Limitations

Several limitations should be considered. First, participants were recruited through community-linked settings in Lisbon. Men who were severely isolated, physically unable to access organizations, experiencing acute mental illness or cognitive impairment, living in institutions, or distrustful of research were less likely to enter the recruitment pathways. Completion of repeated interviews and diaries also selected for availability, sustained consent, and capacity to engage reflectively. The findings are therefore most transferable to community-dwelling older men able and willing to complete an intensive qualitative protocol; they should not be assumed to represent men with the fewest resources or most severe disconnection. In those groups, the balance among autonomy, dependence, communication, and material constraint may differ substantially.

Second, participation required willingness to engage in repeated in-depth interviews and diary activities. Men who completed the study may have had greater reflective capacity, interest in discussing relationships, digital confidence, or availability than other older men. Their accounts cannot be treated as representative of all older men in Lisbon or Portugal.

Third, the concept of unnamed loneliness may have shaped data collection and analysis. Although it was used as a sensitizing rather than predetermined concept, the researcher may have been more attentive to accounts that could be interpreted through loneliness. Reflexive procedures and attention to negative cases reduced but could not eliminate this possibility.

Fourth, repeated interviewing, diary completion, participant-led elicitation, and revisiting earlier accounts may have changed how participants attended to and interpreted relationships, silence, intimacy, and usefulness. Apparent increases in willingness to name loneliness therefore cannot be attributed confidently to naturally occurring change. They may reflect life events, research elicitation, or an interaction between the two. This reactivity is central to the interpretation of the longitudinal findings: the study observed change within a reflective research relationship, not an untouched natural trajectory. At the same time, the possibility that structured reflection can make relational need compatible with competence is analytically informative and warrants prospective study rather than being treated solely as bias.

Fifth, photographs and audio diaries were self-selected representations. They did not provide objective records of participants’ everyday lives. Participants may have documented experiences they considered acceptable, meaningful, or relevant to the research while excluding more private or stigmatized aspects.

Sixth, participants’ accounts of family members, partners, and friends reflected their own perspectives. Including relational partners could have generated a more dyadic understanding, particularly in relation to caregiving, marriage, and family contact. However, doing so would have altered the study’s focus and raised additional confidentiality concerns.

Seventh, the qualitative design does not permit the estimation of the prevalence of unnamed loneliness or statistical comparison among demographic groups. Differences related to age, relationship status, sexual orientation, socioeconomic position, or health should be interpreted as contextual patterns rather than population-level effects.

Finally, the cultural specificity of Lisbon must be considered. Portuguese expectations concerning family responsibility, masculinity, aging, and intergenerational support may differ from those in other countries and from those in rural or less densely populated Portuguese regions. Transferability should therefore be assessed in relation to context rather than assumed.

### 4.12. Directions for Future Research

Future research should examine unnamed loneliness among older men in other Portuguese regions, particularly rural and geographically isolated communities, where mobility, transport, depopulation, and access to services may shape connections differently.

Research should also include men who are underrepresented in community-based studies, including residents of long-term care settings, men with severe disability, migrants, men without stable housing, male caregivers, and men experiencing alcohol-related or other mental-health difficulties.

Dyadic studies could examine how older men’s loneliness is perceived by spouses, adult children, close friends, or caregivers. This would help identify discrepancies between men’s internal experiences and how their well-being is understood by others.

Further research with gay, bisexual, transgender, and other sexual- and gender-minority older men is also needed. Such studies should examine chosen families, minority stress, HIV-related histories, ageism, partnership opportunities, and expectations regarding health and residential care.

Methodologically, future work could compare direct questioning with narrative, visual, and indirect approaches to determine what different methods reveal or obscure. The proposed processes of nonrecognition, linguistic substitution, identity protection, and relational concealment could also be examined quantitatively after careful qualitative conceptual development.

Intervention research should test whether contribution-oriented and identity-affirming programs are more acceptable and effective than services explicitly labeled as loneliness interventions. Outcomes should extend beyond the number of social contacts to include intimacy, belonging, perceived usefulness, purpose, emotional disclosure, and the quality of relationships.

### 4.13. Conclusions

This study suggests that loneliness among older men may remain unrecognized when it is defined too narrowly as living alone, having few contacts, or explicitly stating “I am lonely.” Participants frequently communicated relational distress through boredom, silence, loss of usefulness, absence of intimacy, rigid self-reliance, and the belief that they no longer occupied a meaningful place in other people’s lives.

Unnamed loneliness emerged as a relational and identity-protective process involving nonrecognition, linguistic substitution, identity protection, and concealment. Masculine self-reliance was neither uniformly harmful nor uniformly protective. It could sustain dignity and resilience while also restricting disclosure and access to support.

The findings indicate that strengthening connections requires more than increasing contact. Older men may need relationships and social roles in which they are recognized, expected, emotionally safe, and able to contribute. Clinical and community responses should therefore protect autonomy while expanding opportunities for reciprocity, intimacy, purpose, and meaningful participation.

By attending to the language men use—and to the experiences they communicate without naming—the study offers a more nuanced account of loneliness in later life. It also underscores the importance of asking not only whether an older man has people around him, but whether he feels known, needed, and able to reach others when his life becomes difficult.

## Figures and Tables

**Table 1:** Socio-demographic and contextual characteristics of participants (N = 32).

Characteristic	Category or Statistic	n	%
Age, years	M = 72.4, SD = 5.8, range = 60–83	-	-
Age group	60–69 years	10	31.2
	70–79 years	18	56.2
	≥80 years	4	12.5
Relationship status	Married or partnered	12	37.5
	Widowed	8	25.0
	Divorced or separated	8	25.0
	Never married or currently single	4	12.5
Living arrangement	Living alone	17	53.1
	Living with a spouse or partner	11	34.4
	Living with adult children or other relatives	3	9.4
	Supported community accommodation	1	3.1
Sexual orientation	Heterosexual	26	81.2
	Gay	4	12.5
	Bisexual	2	6.2
Parental status	Had one or more children	25	78.1
	Had no children	7	21.9
Educational attainment	Primary education	6	18.8
	Secondary education	14	43.8
	Higher education	12	37.5
Employment status	Fully retired	28	87.5
	Part-time, occasional, or informal work	4	12.5
Time since retirement ^a^	<5 years	7	25.0
	5–10 years	9	32.1
	>10 years	12	42.9
Self-rated health	Good or very good	9	28.1
	Fair	15	46.9
	Poor or very poor	8	25.0
Chronic health condition	One or more chronic conditions	24	75.0
	No reported chronic condition	8	25.0
Mobility	Reported mobility limitations	11	34.4
	No substantial mobility limitations	21	65.6
Caregiving status	Current primary or regular caregiver	5	15.6
	Not currently a caregiver	27	84.4
Community participation	Regular participation	18	56.2
	Limited or no regular participation	14	43.8
Recent major life transition ^b^	Bereavement	9	28.1
	Major illness or disability transition	8	25.0
	Retirement	7	21.9
	Separation or divorce	4	12.5
Loneliness self-identification at baseline	Explicitly identified as lonely	13	40.6
	Did not identify as lonely	19	59.4
Study completion	Completed all study stages	32	100.0

Note. Percentages may not total 100 due to rounding. ^a^ Calculated among the 28 fully retired participants. ^b^ Participants could report more than one major life transition; therefore, percentages are not mutually exclusive. “Regular community participation” was defined as participation in an organized or informal community, recreational, cultural, religious, educational, or volunteering activity at least twice per month. Loneliness self-identification was based on participants’ explicit use or acceptance of the term lonely during the initial interview, rather than on a standardized loneliness measure.

## Data Availability

The data are not publicly available because the interview transcripts, audio diaries, photographs, and case-level materials contain sensitive and potentially identifying information. De-identified excerpts may be made available from the corresponding author upon reasonable request, subject to institutional ethical approval, data-protection requirements, and the scope of participants’ informed consent.
